# Structural Disruption of Cilia and Increased Cytoplasmic Tubulin in Biliary Atresia—An Exploratory Study Focusing on Early Postoperative Prognosis Following Portoenterostomy

**DOI:** 10.3390/biomedicines13010087

**Published:** 2025-01-01

**Authors:** Patrícia Quelhas, Rui Oliveira, Carlos Kieling, Sandra Vieira, Jorge dos Santos

**Affiliations:** 1 Faculty of Health Sciences, Health Science Investigation Center of University of Beira Interior (CICS-UBI), 6200-506 Covilhã, Portugal; patriciaasquelhas@gmail.com; 2 Coimbra Institute for Clinical and Biomedical Research (iCBR), Area of Environment, Genetics and Oncobiology (CIMAGO), Faculty of Medicine, University of Coimbra, 3000-548 Coimbra, Portugal; ruipedrocoliveira@hotmail.com; 3 Pathology Department, Centro Hospitalar e Universitário de Coimbra, 3000-075 Coimbra, Portugal; 4 Germano de Sousa-Centro de Diagnóstico Histopatológico CEDAP, University of Coimbra, 3000-377 Coimbra, Portugal; 5 Unidade de Gastroenterologia e Hepatologia, Hospital de Clínicas de Porto Alegre (HCPA), Porto Alegre 90035-903, Brazil; ckieling@hcpa.edu.br (C.K.); smvieira@hcpa.edu.br (S.V.); 6 Department of Pediatrics, Universidade Federal do Rio Grande do Sul (UFRGS), Porto Alegre 90010-150, Brazil; 7 Programa de Transplante de Fígado Pediátrico, Hospital de Clínicas de Porto Alegre (HCPA), Porto Alegre 90035-903, Brazil

**Keywords:** biliary atresia, primary cilia, cytoplasmic tubulin, hypoxia, native liver survival

## Abstract

**Introduction:** Biliary atresia (BA) is a progressive hepatobiliary disease in infants, leading to liver failure and the need for transplantation. While its etiopathogenesis remains unclear, recent studies suggest primary cilia (PC) disruption plays a role. This study investigates correlations between PC and cytoplasmic tubulin (TUBA4A) alterations with hypoxia in patients with the isolated form of BA, focusing on native liver survival. **Methods:** Using qualitative and quantitative digital image analysis of immunofluorescence-stained liver samples, we assessed PC and TUBA4A features correlating these findings with HIF-1α nuclear positivity, clinical–laboratory data, and early native liver survival. Liver samples from fourteen BA patients and six controls with another liver disease were analyzed by digital image analysis, with data evaluated using Spearman’s correlation and independent *t*-tests. **Results:** HIF-1α positivity in cholangiocytes was observed in 42.8% of BA patients. While the PC ratio per biliary structure (cilia ratio status, CRs) was similar between BA patients and controls, PC length was decreased in BA patients. Cytoplasmic TUBA4A levels were elevated in BA patients. CRs positively correlated with lower cytoplasmic TUBA4A expression and was higher in patients without HIF-1α nuclear positivity. Reduced cilia length correlated with higher bilirubin levels at portoenterostomy. Predictors of early poor prognosis (death or need for transplantation until 1 year of life) included HIF-1α positivity, elevated direct bilirubin levels, decreased cilia length, PC bending, and increased TUBA4A expression. **Conclusions:** Reduced PC length, PC bending, and increased intensity of cytoplasmic TUBA4A expression occur in the isolated BA clinical type and negatively impact the early prognosis after post-portoenterostomy. These findings suggest the existence of a disruption in the tubulin transport between cytoplasm and PC. The detrimental effect of HIF-1alpha pathway activation over early native liver survival was confirmed, although independently from PC or cytoplasmic tubulin features.

## 1. Introduction

Biliary atresia (BA) is a rare, life-threatening disease that starts in infancy and comprises a progressive obstructive cholangiopathy expanding from the extrahepatic biliary tract to the intrahepatic biliary tree [1,2]. In BA, there is an early extensive ductular reaction and associated fibrosis, which unleashes cirrhosis and chronic liver failure, leading to the need for liver transplantation (LTx), or death, until the second year of life. Presently, the only treatment for BA is a portoenterostomy developed by Morio Kasai. If timely performed, Kasai portoenterostomy enables 5- and 10-year survival rates of around 75% and 67%, respectively [3,4]. Whenever portoenterostomy is ineffective, postoperative native liver survival (NLS) does not surpass 2 years of life. Around 22% of BA patients reach 30 years of life without LTx given the increased NLS rates after portoenterostomy presently obtained [5]. The occurrence of different clinical forms of BA, including the most frequent variant, called isolated BA, suggests the existence of diverse etiopathogenetic mechanisms for the development of the disease [6,7]. The etiology of BA seems to result from viral infection, toxins, immunogenetic factors, and defects in embryogenesis. Genetic and epigenetic predispositions, associated with environmental factors affecting the mother, are potential triggers for BA [8,9]. Concerning the effects of environmental factors on the mother, a recent large case-control study indicates an association between prenatal maternal intestinal or genitourinary tract infection and the occurrence of BA in the offspring [10].

Our group, after confirming a progressive medial layer thickening of hepatic arterial branches—suggestive of vascular remodeling in patients with isolated BA [11,12]—investigates the role of hypoxia-ischemia affecting the biliary structures as a putative cause of the cholangiopathy. We detected histopathologic alterations and specific gene expression profiles in *VEGF*, Angiopoietins, their receptors, and HIF-1alpha (HIF-1α) pathways [13,14,15]. Later, we uncovered through immunohistochemistry a strong HIF-1α positivity in intrahepatic biliary structures of at least 35.7% of patients with isolated BA, involving the portal tracts, portal–parenchymal interfaces, and fibrovascular septal and subcapsular areas [16]. Since the HIF-1α pathway was found to be activated in cholangiocytes within the portal–parenchymal interface, where the hepatobiliary progenitor cells’ niche is located, it is conceivable that hypoxia and other processes involved with HIF-1α activation affect liver regeneration and fibrogenesis [17]. These consecutive studies suggest that at least a subset of BA patients present a cholangiopathy attributable to hypoxia [6,18]. A relevant investigation involving systems analysis integrating high-throughput biological data confirmed a central role of hypoxia and HIF-1α pathway activation in the development of BA [19]. One recent study confirmed the presence of hepatic artery medial layer thickening in BA and its detrimental effects on the liver, attributable to hypoxia, relating the vascular disruption with a disorder of the Notch3/Hey1 pathway [12]. Another study, using hypoxic injury over fetal/neonatal extrahepatic bile ducts (EHBDs) in sheep, revealed that hypoxia causes luminal narrowing and formation of biliary mucous plugs, a process which, after completion of the fetal/neonatal period, transitions into the adult scarring/fibrosing process, characteristic of BA [20]. Thus, hypoxic injury represents a putative mechanism for the pathophysiology of BA.

Primary cilia (PC) are solitary luminal projections present in the cholangiocyte apical membrane that function as tunable sensing organelles and whose permanent loss leads to disease. The structural base of PC, as that of cytoplasmic microtubules, consists of doublets of alpha- and beta-tubulin dimers assembled as closed cylinders that collectively are referred to as axoneme [21,22]. Sensory and signaling functions of PC are essential for embryonic development, and gene variants that cause the loss of PC result in ciliopathies. Primary cilia act as chemoreceptors and mechanoreceptors in the biliary system, likely involved in cell proliferation, senescence, activation of the progenitor cell compartment, regeneration, and embryonic developing mechanisms [22,23]. Concerning BA, PC abnormalities seem to occur in BA patients with or without associated extrahepatic anomalies [24,25]. A reduced amount of PC in intrahepatic biliary structures in the livers of patients with BA seems to worsen the postoperative prognosis after portoenterostomy [26]. However, as far as we know, alterations in the behavior of the cytoplasmic microtubule Tubulin-alpha 4A (TUBA4A)—which are known to be associated with PC disorders [27,28]—have not yet been analyzed in BA.

Given the described alterations of cholangiocyte PC in biliary atresia patients and their effects on the disease prognosis in the scarce references from the literature [26,27,28,29], we performed this study using adequate quantitative image analysis of fluorescent stained samples to (1) confirm specific cholangiocyte PC characteristics, including deciliation, PC length, and bending in patients with the isolated form of BA; (2) investigate the effects of ciliary alterations over the 1-year native liver survival after portoenterostomy. In addition, considering the recognized effects of hypoxia over PC dysfunction and the previous evidence of HIF-1alpha activation in the biliary epithelium of a subset of BA patients, we aimed to correlate the HIF-1α pathway activation in cholangiocytes of BA patients with the primary cilia features and the 1-year native liver survival. Finally, since the intensity of cytoplasmic TUBA4A expression has not been evaluated in BA patients, we intended to correlate this variable with PC features and the 1-year native liver survival.

## 2. Materials and Methods

This histopathological study analyzed selected microanatomic structures in the liver of each patient included in a convenience sample of the isolated BA group, compared to controls with another disease, to identify specific histopathological (immunofluorescence and morphometric) behavior patterns involving the variables of interest (Table 1 and Table 2, and Figure S2 from Supplementary Materials).

### 2.1. Patients and Samples

All the patients enrolled in this study underwent exploratory laparotomy between 2005 and 2018 as part of the diagnostic workup for neonatal cholestasis at Hospital de Clínicas de Porto Alegre, Brazil. The inclusion criteria for patients in this study comprised infants with isolated BA and controls with intrahepatic neonatal cholestasis of comparable age—submitted to exploratory laparotomy with trans-operative cholangiogram in the process of diagnostic investigation—and patients with BA that maintained postoperative follow-up until the end of the study or died. Exclusion criteria comprised infants with clinical types of BA different from the isolated form, absence of histopathological confirmation of BA through the analysis of biliary remnants in the porta hepatis, and BA patients that did not fulfill the follow-up until the completion of the study.

The study group consisted of fourteen patients with BA for whom exploratory laparotomy preceded portoenterostomy, and the control group included six patients with intrahepatic cholestasis (IHC) for whom this invasive diagnostic procedure was necessary to rule out BA (Table 2). The diagnosis of BA was confirmed by both exploratory laparotomy with intraoperative cholangiogram and postoperative bile duct evaluation in porta hepatis. Isolated BA was characterized by the absence of biliary atresia splenic malformation (BASM), extrahepatic bile duct cysts, or positive IgM serology for cytomegalovirus. Clinical–laboratory, molecular, and histological criteria defined the IHC group. The final diagnoses of IHC controls included idiopathic neonatal cholestasis (n = 3), alpha-1 antitrypsin deficiency (n = 1), cytomegalovirus hepatitis (n = 1), and parenteral nutrition-associated cholestasis (n = 1). During the exploratory laparotomy, a wedge liver biopsy was excised from the hepatic segment IV in each patient. In those patients with image evidence of choledochal anatomical blockade, the porta hepatis was extracted. Liver samples were paraffin-embedded and used for immunofluorescence analysis. Preoperative laboratory tests were conducted for all the patients, and the surgery was performed if serum hemoglobin levels were adequate for a safe procedure. The duration and type of anesthesia did not differ between patients and controls, and there was no evidence of intraoperative hypoxia in any of the infants studied.

### 2.2. Clinical–Laboratory Data Collection

Clinical and laboratory data were prospectively collected and stored in a secure databank (Table 2). Concerning the laboratory tests for comparisons between groups, the serum bilirubin level was selected as an indicator of NLS after portoenterostomy.

### 2.3. Immunofluorescence Staining

Liver paraffin-embedded samples were microtome-sectioned into 5 µm slices, deparaffinized with xylene, and rehydrated in decreasing ethanol concentrations (100%, 95%, and 70%) and distilled water. Since the samples were fixed in formalin, and formaldehyde residues are autofluorescent in the green spectrum, a treatment with 0.02% Methanol–Peroxidase was performed for 30 min at room temperature under constant agitation. Antigen retrievals were executed using Sodium citrate buffer (10 mM Sodium citrate, 0.05% Tween 20, pH 6.0) in a pressure cooker for 6 min after boiling.

Samples were permeabilized with PBS 1% Tween for 15 min at room temperature and blocked with PBS 10% Fetal Bovine Serum for 1 h at room temperature. Immunostaining with recombinant HIF-1α (Novus Biological, AF1935; 1:100) and TUBA4A (Santa Cruz Biotechnologies, Sc-23950; 1:100) antibodies was carried out at room temperature for 3 h. Secondary antibodies (Alexa-568—A175474, Abcam; Alexa-647—Ab150115, Abcam, 1:20,000) were stained for 1 h at room temperature, and nuclei were stained using DAPI for 10 min at room temperature. A human gallbladder specimen was used as a positive control for the HIF-1α signal, and a human testicle for the TUBA4A signal (Figure S1, Supplementary Materials).

### 2.4. Image Analysis

#### 2.4.1. Qualitative Analysis

An initial qualitative analysis of TUBA4A and HIF-1α labeling was performed by the authors RCO and JLS—both with expertise in liver histopathology—first separately and then under consensus. Concerning both HIF-1α and TUBA4A, the distribution of their staining was observed in the cholangiocytes according to our previous study [16]; this included within the portal tracts, at the portal–parenchymal interface, in fibrovascular septa and subcapsular areas, as well as in the ductular reaction spreading to the parenchyma. HIF-1α positivity in cholangiocytes was defined as the strong nuclear positivity exclusively in biliary structures. Although HIF-1α positivity occurred in hepatocytes with varying parenchymal zonal locations and intensities, we did not analyze these data for the present study. In the case of TUBA4A, the location of positivity was classified as cytoplasmic, apical, and ciliary.

#### 2.4.2. Quantitative Analysis

##### HIF-1α Nuclear Positivity Analysis

The analysis of fluorescence images was conducted using QuPath 0.4.4 software [29], following a standardized protocol to ensure reproducibility. To define a positivity threshold for HIF-1α staining, a positive control (human gallbladder, large cholangiocytes) was used (Figure S1A, Supplementary Materials). Nuclei were identified and isolated using the DAPI channel, and their intensities in the HIF-1α channel were quantified using the ”Positive Cell Detection” tool in QuPath (“Intensity Threshold Parameters: nucleus DAPI mean”). Subsequently, quantifications (mean intensity per nucleus) were exported (QuPath: ”measure—show detection results”), and a histogram of the average intensity per nucleus was generated. This histogram revealed two distinct peaks corresponding to positive and negative populations, with the threshold set at the midpoint between these peaks to differentiate the two groups.

Portal tracts, portal–parenchymal or septal–parenchymal interfaces, and areas of ductular reaction extending externally to parenchymal zone 1 were selected as regions of interest (ROIs). These areas were manually delineated using QuPath’s annotation tools. The ”Positive Cell Detection” tool in QuPath was then applied to count HIF-1α—positive nuclei. Cells were categorized into two groups, negative or positive, based on the predefined threshold.

##### TUBA4A Analysis—Cytoplasmic Expression

Cytoplasmic expression for TUBA4A was conducted using QuPath 0.4.4 software. To define the positivity threshold, a positive control for TUBA4A staining (human testicle) was used (Figure S1B, Supplementary Materials).

Bile ducts, ductules, and interface structures were selected and, through the ”Positive Cell Detection” tool in QuPath (“Intensity Threshold Parameters: cytoplasm TUBA4A mean”), positive cells in the cytoplasm were counted and divided into 2 categories: low expression and high expression for TUBA4A. Cells were isolated using DAPI, and their intensities in the TUBA4A channel were quantified. Subsequently, quantifications (mean intensity per cell) were exported (QuPath: ”measure—show detection results”), and the histogram of the average intensity per cell was observed, revealing two peaks (positive and negative). The value between these peaks was set as the positivity threshold and subsequently divided into groups of low and high expression.

##### TUBA4A Analysis—Cilia Quantification

For cilia analysis, at least 5 images per sample were acquired with a 40× magnification (Axio Imager Z2 Microscope, Zeiss, Germany). Subsequently, the CiliaQ v0.1.4 plugin [30] from the image analysis program ImageJ was used to quantify and calculate the length and bending of cilia in cholangiocytes per image. The plugin presents three steps for image segmentation and processing. In the first step of the workflow, using the CiliaQ v0.1.2 Preparator, the Hysteresis Threshold was applied to segment the fluorescence channel labeling TUBA4A (cilia), and in the second step (CiliaQ v0.0.3 Editor), everything within the threshold that was not cilia (labeling debris) was discarded. Finally, in the CiliaQ v0.1.4 step, cilia were quantified, and the length and bending of each cilium were measured. Given that in BA samples there is a larger number of biliary structures—mainly in the portal–parenchymal interface, ductular reaction, and fibrovascular septa—to calculate the total number of cilia per sample, a ratio of the total number of cilia to the total number of ducts/ductules analyzed, called cilia ratio status (CRs), was computed (Figure S3, Supplementary Materials).

### 2.5. Correlation of Image Analysis Data with Postoperative Outcomes: Death, LTx, and Native Liver Survival After Portoenterostomy

To evaluate the impact of cholangiocyte cilia characteristics (presence/absence, length, and bending) and cytoplasmic TUBA4A expression on the outcomes following portoenterostomy, we focused on the 1-year native liver survival rate as the primary outcome measure. Spearman’s correlation analysis was conducted to explore associations between the clinical data and image analysis results to provide insights into how these factors may influence the early postoperative prognosis.

### 2.6. Statistics

Quantitative variables were expressed as individual data points and mean ± SD and categorical data were described as frequencies and percentages. The Shapiro–Wilk and Levene tests were performed to assess the normality of the quantitative variables, and all were found to follow a normal distribution. Therefore, an independent Student’s *t*-test was used to compare the groups. Spearman’s correlation was applied to correlate quantitative and qualitative variables. The statistical power of significant results was calculated using Cohen’s d, with data interpretation according to the Supplementary Materials, Table S1. A two-tailed *p*-value < 0.05 was accepted as significant. Statistical Package for the Social Sciences (SPSS, version 27.0, IBM Corp., Armonk, NY, USA) was used for data processing and statistical analysis, and GraphPad Prism 8 for graph images and processing.

### 2.7. Ethics

This study was approved by the Research Ethics Committee at the Hospital de Clínicas de Porto Alegre (HCPA), Brazil (project: arteriopathy in biliary atresia and its relation with the postoperative prognosis after portoenterostomy, number 130030) and performed by the ethical standards outlined in the Declaration of Helsinki. Informed consent for using biological specimens and clinical data of the infants was obtained from parents or legal guardians. Information from the databank used in this study was pseudonymized to protect the privacy of patients and families. The liver samples were stored in the “Biorepository of Biological Specimens in Pediatric Hepatology” of the Center of Experimental Research (CPE) of Hospital de Clínicas de Porto Alegre (HCPA), respecting ethical and methodological aspects. This study was approved by the Board of the Foundation for Science and Technology (FCT) under the European/Portuguese collaboration project Portugal 2020 (title: the role of ischemic cholangiopathy in conditions of hepatic dysfunction, project number 02/SAICT/2017—IsChoHep FCT FEDER).

## 3. Results

### 3.1. Clinical and Laboratory Data of the BA Patients

The liver tissue samples of 20 patients were assessed: 14 with BA and 6 with IHC (control group). The demographic and clinical characteristics of patients and controls are presented in Table 2. At the time of surgery, the age ranged from 32 to 110 days (mean 62.7 ± 21.4) in BA patients and from 35 to 78 days (mean 52.7 ± 17.1) in controls, with no significant difference between the groups. During the total follow-up period (2005 to 2018), five patients underwent LTx and five patients died, four of them without having undergone LTx. Age at LTx ranged from 3.8 to 85.7 months (median 46.8 months). Age at death with the native liver ranged from 5.5 to 9.5 months (median 8.3 months), reflecting a native liver survival of less than one year. Nine BA patients survived until the end of the study, five of them with their native liver. Regarding bilirubin serum levels at portoenterostomy, total bilirubin (TB) ranged from 4.6 to 19.1 mg/dL (mean 9.6 ± 4.1), and direct bilirubin (DB) ranged from 3.5 to 14.3 mg/dL (mean 7.1 ± 3.1).

### 3.2. Detection and Immunolocalization of HIF-1α and TUBA4A by Qualitative Method

**Figure 1:**
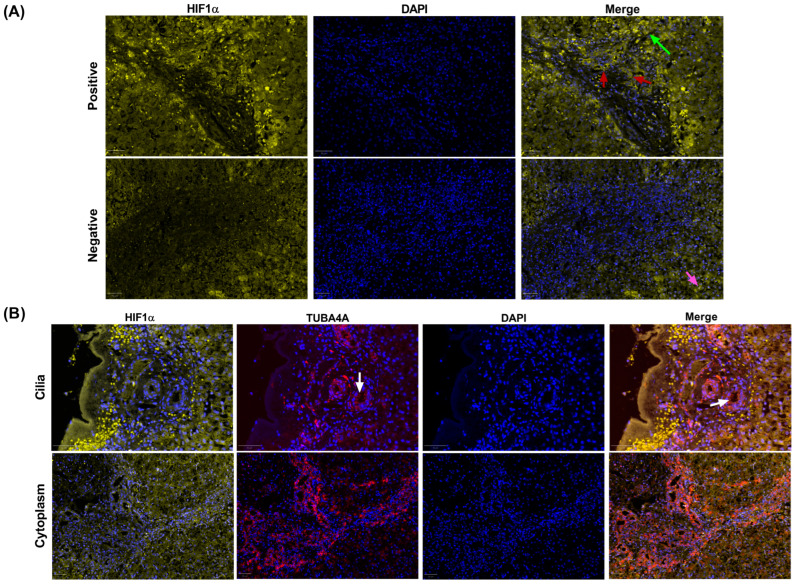
HIF-1α nuclear activation status in cholangiocytes. (**A**) Above (positive): HIF-1α nuclear positivity in the liver of a BA patient. HIF-1α positivity was observed in cholangiocytes of the portal tracts, including interlobular bile ducts (red arrows); the portal–parenchymal interface (green arrow); ductular reaction expanding outwards to parenchymal zone 1; and fibrovascular septa, including intra-septal and septal interface ductular reactions. Below (negative): a patient with idiopathic neonatal cholestasis. Hepatocytes also exhibited HIF-1α nuclear positivity (pink arrow) with variable intensity according to the zonal distribution (pericentral or periportal), in both BA and control samples, independent of the biliary HIF-1α positivity status. Images magnification: 20×. Blue: DAPI staining; Yellow: HIF-1α staining. (**B**) Above (cilia): representative image from a BA sample with TUBA4A staining in cholangiocytes of the portal tracts with ciliary configuration (white arrows). Below (cytoplasm): representative image from BA sample with TUBA4A staining in cytoplasm of cholangiocytes in the portal tract and portal–parenchymal interface. Images magnification: 20×. Blue: DAPI staining; Yellow: HIF-1α staining; Red: TUBA4A staining.

HIF-1α nuclear positivity was not detected in any biliary structures in the IHC controls (Figure 1A). In contrast, five out of fourteen (35.7%) patients with BA showed HIF-1α nuclear positivity in cholangiocytes of the portal tracts, portal–parenchyma interface, ductular reaction expanding outwards to parenchymal zone 1, fibrovascular septa, and subcapsular area (Figure 1A).

In BA samples, TUBA4A positivity was observed in the cytoplasm and/or apical membrane of cholangiocytes, in the last case, with or without associated ciliary structures (Figure 1B). Cytoplasmic TUBA4A positivity was noted in cholangiocytes located in the portal tracts, portal–parenchymal interface, ductular reaction expanding outwards to parenchymal zone 1, fibrovascular septa, and subcapsular area (Figure 1B).

#### Qualitative vs. Quantitative Analysis

Qualitative and quantitative methods were used to characterize HIF-1α nuclear positivity. According to the quantitative method, a higher number of cases (six out of fourteen; 42.8% of cases) presented HIF-1α nuclear positivity in cholangiocytes than through the qualitative method (five out of fourteen; 35.7% of cases), showing a small variability between the two evaluation processes.

### 3.3. Quantitative Analysis of Ciliary Characteristics in Cholangiocytes of Biliary Atresia Patients

**Figure 2:**
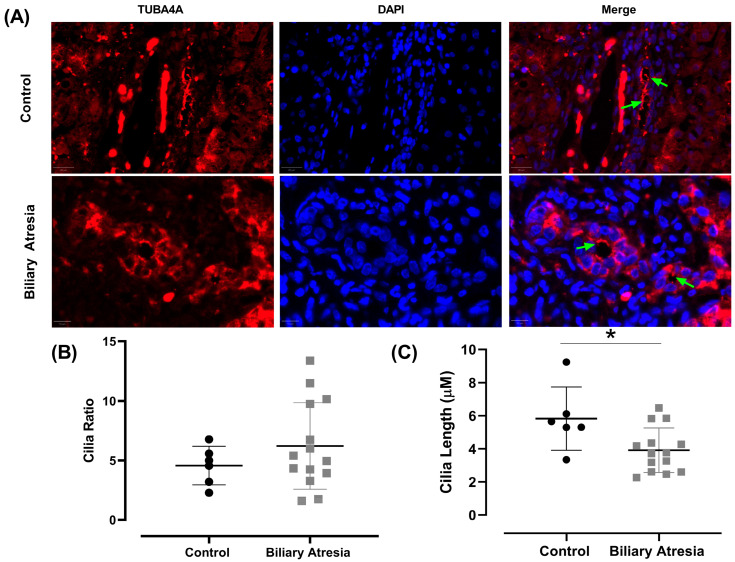
Comparison of cholangiocyte primary cilia features in biliary atresia and controls. (**A**) Representative images showing the differences between controls and BA. Green arrows indicate the cilia of cholangiocytes. Images Magnification: 40×. Blue: DAPI staining; Red: TUBA4A staining. (**B**) Comparison of cilia ratio status between controls and BA. (**C**) Comparison of cilia length between controls and BA (Student’s *t*-test, * *p* = 0.010, *d* = 1.249). Dots represent individual data points. Horizontal lines indicate the mean, and vertical lines represent the SD.

When evaluating the CRs across the different assessed areas, no difference was observed between the CRs of BA patients versus controls (Figure 2B). However, regarding primary cilia length, the cilia in the biliary structures of BA patients were notably shorter (Figure 2C).

**Figure 3:**
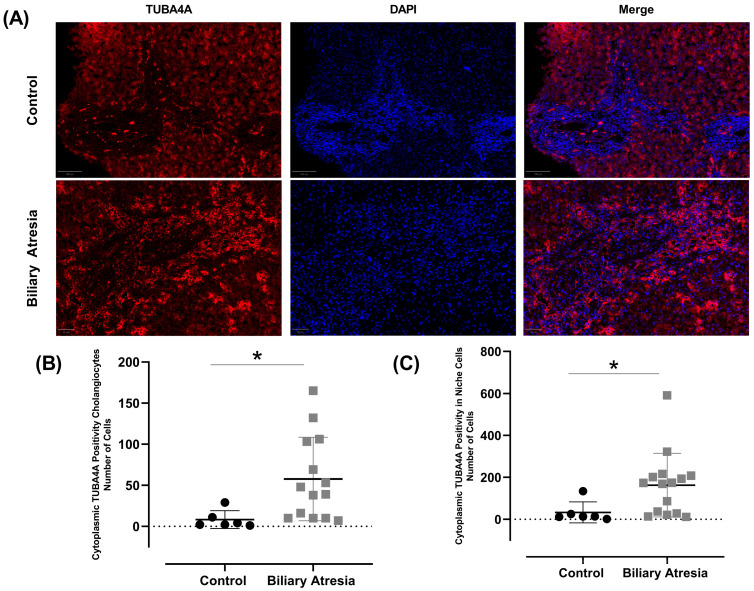
Comparison of the cytoplasmic TUBA4A positivity in cholangiocytes and niche cells. (**A**) Representative images showing the differences between controls and BA. Images Magnification: 40×. Blue: DAPI staining; Red: TUBA4A staining. (**B**) Comparison of cytoplasmic TUBA4A cholangiocyte positivity between controls and BA (*t*-test, * *p* = 0.005, *d* = 0.953). (**C**) Comparison of cytoplasmic TUBA4A positivity in niche cells between controls and BA (*t*-test, * *p* = 0.045, *d* = 0.924). Dots represent individual data points. Horizontal lines indicate the mean, and vertical lines represent the SD.

A significant increase in cytoplasmic TUBA4A expression was observed in BA patients compared to the controls (Figure 3A). TUBA4A expression intensity was higher in biliary structures within the portal tracts and along the portal–parenchymal interfaces (Figure 3B,C). The cilia ratio status presented a strong positive correlation with a lower cytoplasmic TUBA4A expression (Table 3).

### 3.4. Relationship Between HIF-1α Expression and Ciliary Characteristics in BA

**Figure 4:**
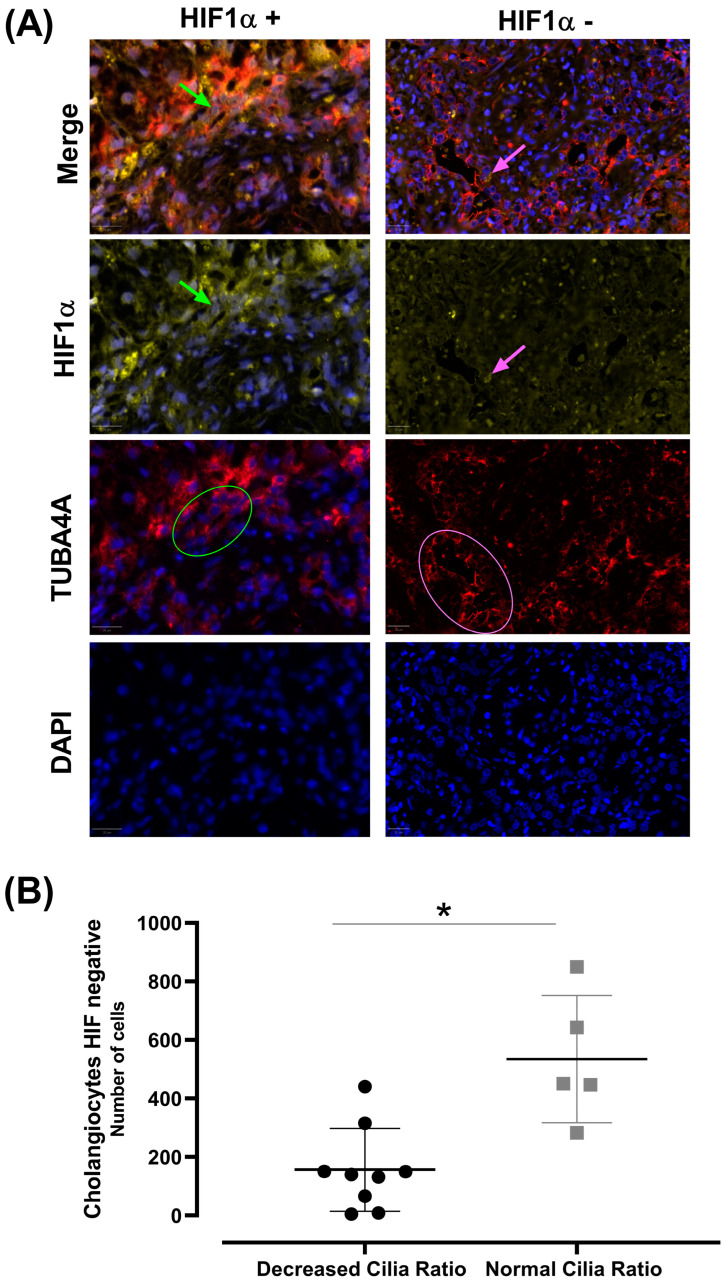
HIF-1α influence over cholangiocyte cilia. (**A**) Representative images demonstrating the influence of HIF-1α positivity on cholangiocyte cilia. The images in the left column display a biliary duct positive for HIF-1α (indicated by green arrows) with no visible cilia in the duct (highlighted by the green circle). In contrast, the right column shows a biliary atresia sample with HIF-1α-negative cholangiocytes (indicated by pink arrows) where the cilia in the ducts are clearly evident (highlighted by the pink circle). Images magnification: 40×. Blue: DAPI staining; Yellow: HIF-1α staining; Red: TUBA4A staining. (**B**) Considering the quantitative evaluation of the extent of HIF-1α nuclear expression and the cilia ratio status in the sample of BA, a significant increase in cilia ratio status when the amount of HIF-1α negative cells is higher is observed (Student’s *t*-test, * *p* < 0.001, *d* = 2.216). Dots represent individual data points. Horizontal lines indicate the mean, and vertical lines represent the SD.

Regarding the colocalization of HIF-1α and TUBA4A in BA samples, the presence of HIF-1α nuclear positivity in cholangiocytes often correlated with a cytoplasmic pattern of TUBA4A positivity without identifiable PC. Conversely, HIF-1α negativity was frequently associated with identifiable PC structures in the apical membrane of cholangiocytes. The staining of HIF-1α and TUBA4A was analyzed to understand the behavior of cilia under hypoxic conditions. Cholangiocytes with lower levels of HIF-1α expression exhibited increased TUBA4A staining, indicating a higher rate of PC presence (Figure 4A). Due to the high variability in CRs, the data were divided into two groups based on the median value: normal CRs and decreased CRs (Figure 4B). This division allowed for a clearer understanding of the relationship between HIF-1α expression and cilia presence. Considering the quantitative evaluation of the extent of HIF-1α nuclear expression and the cilia ratio status in the sample of BA, a significant increase in cilia ratio status was observed when the amount of HIF-1α negative cells increased.

### 3.5. Effects of HIF-1α as Well as Ciliary and Cytoplasmic TUBA4A Morphometric Features on the Clinical–Laboratory Status and Native Liver Survival

Table 3 presents the results of the correlations between quantitative and qualitative clinical variables and native liver survival.

#### 3.5.1. Correlations Between Clinical–Laboratory, Image Morphometric, and Outcome Variables

Concerning BA patients, some relevant correlations occurred between the clinical–laboratory data and quantitative morphometric results. Age at portoenterostomy did not present correlations, neither with HIF-1α positivity nor with TUBA4A expression in cholangiocytes. However, cilia length presented negative correlations with total bilirubin (ρ = −0.665; *p* = 0.013) and direct reacting bilirubin (ρ = −0.758; *p* = 0.022).

Concerning the statistical relationships among the different image morphometric variables, lower TUBA4A cytoplasmic expression presented a strong positive correlation with cilia ratio status (ρ = 0.890; *p* < 0.001). Regarding the outcome variables, early LTx (until 1 year of life) was positively correlated with direct-reacting bilirubin serum levels at portoenterostomy (ρ = 0.624; *p* = 0.023) and negatively correlated with cilia length (ρ = −0.802; *p* = 0.001). On the other hand, early death (until one 1 year of life) showed moderate positive correlations both with HIF-1α nuclear positivity (ρ = 0.692; *p* = 0.009) and higher cytoplasmic TUBA4A expression (ρ = 0.626; *p* = 0.022) in cholangiocytes.

Confirming our previous immunohistochemical findings [16], HIF-1α nuclear positivity correlated with early death rates (ρ = 0.692; *p* = 0.009).

#### 3.5.2. Native Liver Survival

**Figure 5:**
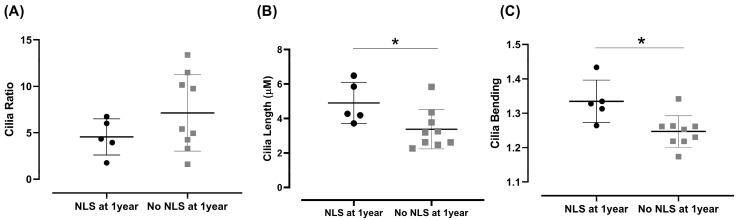
Correlations between native liver survival within 1 year after portoenterostomy and morphometric primary cilia features in the sample of BA patients. (**A**) Comparison of cilia ratio status between BA patients with and without NLS. (**B**) Comparison of cilia length between BA patients with or without NLS (*t*-test, * *p* = 0.018, *d* = 1.317). (**C**) Comparison of cilia bending between BA patients with or without NLS (*t*-test, * *p* = 0.005, *d* = 1.694). Dots represent individual data points. Horizontal lines indicate the mean, and vertical lines represent the SD.

Concerning the correlations between the 1-year NLS after portoenterostomy and the PC features in BA, patients presenting decreased PC length and decreased PC bending either died or needed liver transplantation within 1 year after portoenterostomy (Figure 5). These results suggest that specific alterations in PC morphology are associated with poorer prognosis in BA patients after portoenterostomy.

**Figure 6:**
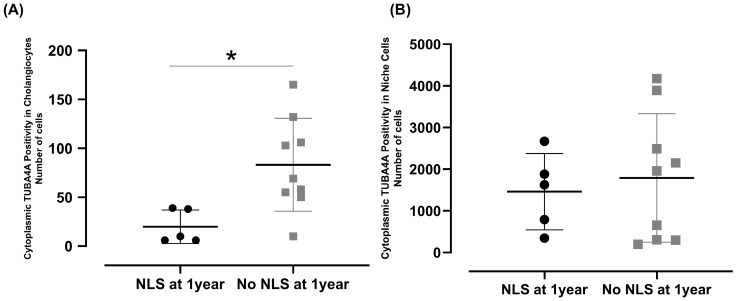
Effects of TUBA4A expression in the cytoplasm of cholangiocytes within the portal tracts and along the portal–parenchymal interface over 1-year rates of native liver survival after portoenterostomy. (**A**) Increased cytoplasmic TUBA4A expression in cholangiocytes was associated with non-achievement of 1-year native liver survival (*t*-test, * *p* = 0.020, *d* = 1.005). (**B**) Difference between TUBA4A cytoplasmic positivity in niche cells regarding native liver survival. Dots represent individual data points. Horizontal lines indicate the mean, and vertical lines represent the SD.

A significant difference concerning the cytoplasmic TUBA4A expression in cholangiocytes occurred between BA patients with and without NLS until 1 year of life (Figure 6A). Increased cytoplasmic TUBA4A expression in cholangiocytes was associated with non-achievement of 1-year NLS (Figure 6A). These findings suggest that higher levels of cytoplasmic TUBA4A in cholangiocytes correlate with poorer outcomes in BA patients.

## 4. Discussion

### 4.1. Clinical Data of the BA Patients for a Prognostic Evaluation

Regarding the clinical data of BA patients under study, given the small sample available, we were not able to evaluate the 6-month NLS after portoenterostomy. However, during the full follow-up period (2005 to 2018), 7 out of 14 (50%) BA patients died and/or were transplanted in the first year of life, which we consider an early bad prognosis. One of these patients (BA10) with an early bad prognosis underwent LTx at 114 days of life and died 8 days later due to systemic complications of liver cirrhosis and chronic liver failure. Four of the patients with early bad prognosis died with their native liver. Three other patients were transplanted and/or died after two years of life.

### 4.2. Detection and Immunolocalization of HIF-1α and TUBA4A

In this study, according to the qualitative evaluation, 35.7% of BA patients showed HIF-1α nuclear positivity in the cholangiocytes of portal tracts, portal–parenchymal interfaces, ductular reaction expanding outwards to parenchymal zone 1, fibrovascular septa, and subcapsular area, whereas HIF-1α positivity was absent in the biliary epithelium of all the controls (Figure 1A). However, the quantitative analyses of HIF-1α positivity in cholangiocytes showed a higher extent of HIF-1α positivity by using the QuPath 0.4.4 software. Six out of fourteen BA cases (42.8%), and not only 35.7%, presented HIF-1α activation in the intrahepatic biliary epithelium, suggesting that the quantitative method has higher sensitivity rates for HIF-1α detection in immunofluorescence-stained samples.

These findings agree with previous studies published by our team [11,13,14,16,17,31,32] in that, at least in a subset of patients, hypoxia-ischemia seems to have a pathophysiological role in BA, corresponding to an ischemic cholangiopathy [18]. Recently, it was demonstrated that the HIF-1α pathway plays a central molecular role in the development of BA [19]. Chang et al. (2024) confirmed that a precocious hepatic arterial medial layer thickening in BA, apparently associated with the Notch3/Hey1 pathway, leads to peribiliary hypoxia [12]. Another group, using a model of hypoxic injury over fetal extrahepatic bile ducts in sheep [20], endorsed that hypoxic injury over fetal/neonatal extrahepatic bile ducts activates a fetal wound healing program and leads to extrahepatic biliary obstruction. A recent epidemiological genetic publication evaluated 811 BA cases in a genome-wide association study, and observed a strong correlation of BA with genes involved in planar polarity, which integrates ciliary dysgenesis and abnormal vascular and biliary epithelial cell development [33].

Concerning tubulin, TUBA4A positivity was observed in the cytoplasm and/or apical membrane of cholangiocytes, occasionally with a ciliary configuration (Figure 1B), both in BA and controls. In BA, cytoplasmic TUBA4A positivity was noticed in cholangiocytes located in the portal tracts, portal–parenchymal interface, ductular reaction expanding outwards to parenchymal zone 1, fibrovascular septa, and subcapsular area (Figure 1B).

### 4.3. Quantitative Analysis of Ciliary Characteristics in Cholangiocytes of Biliary Atresia Patients

Concerning the CRs, unlike previous studies [24,26], our morphometric evaluation did not find a difference between BA patients and controls (Figure 2B). However, a remarkable difference was observed concerning cilia length, being notably shorter in BA (Figure 2C). Cilia length showed significant correlations with clinical and laboratory indicators of cholestasis severity, including total and direct bilirubin serum levels. The presence of PC and their correct structure and functioning indicate the ability of cholangiocytes to detect and respond to extracellular stimuli [34] aiming to regulate fundamental signaling pathways [35].

In this context, PC length is an important variable concerning ciliary function [22]. Cilia length is maintained by the addition or removal of tubulin at the tip of PC. Protein synthesis does not occur in PC, and cilia growth involves the import of tubulin monomers from the cytoplasm into the cilium through tightly regulated processes. Changes in cilia length represent a significant alteration in ciliary volume, and altered molecular concentrations within the cilium are associated with processes that affect PC growth [22]. Genetic or environmental factors can lead to reduced cilia length and the development of ciliopathies [36,37,38].

Given that axoneme length depends on the transit of molecules, including tubulin, between the cytoplasm and cilium, alterations in the microtubule network within the cytoplasm can lead to alterations in the levels of soluble tubulin within PC [27]. Microtubule cytoplasmic stabilization reduces the pool of soluble tubulin and, therefore, may erode the distal tip of the axoneme [38], leading to decreased cilia length or even deciliation.

Additional information obtained in this study was evidenced by the significant increase in cytoplasmic TUBA4A expression in BA patients compared to the controls (Figure 3). Increased TUBA4A expression was particularly evident within the portal tracts and along the portal–parenchymal interfaces. Table 3 shows that higher CRs presented a very strong positive correlation with low or negative cytoplasmic TUBA4A expression (ρ = 0.890; *p* < 0.001).

Importantly, elevated cytoplasmic TUBA4A expression was correlated with increased mortality in BA patients (ρ = 0.626; *p* = 0.022), Table 3. Concerning deciliation, which can be identified in the present study through the variable CRs, it is caused by environmental stresses, including hypoxia [37].

### 4.4. Relationship Between HIF-1α Expression and Ciliary Characteristics in BA

Regarding the colocalization of HIF-1α and TUBA4A in BA samples, in the qualitative analysis, we observed an inverse association between HIF-1α positivity and the presence of PC at the luminal membrane of cholangiocytes, both in biliary cells of portal tracts and at other microanatomic liver areas (Figure 4B). The quantitative analysis of the ciliary characteristics showed that biliary cells without hypoxic characteristics presented preservation of PC, suggesting that hypoxia, indicated by HIF-1α positivity, may negatively impact the formation or maintenance of PC. As previously described, hypoxia constitutes an environmental stress that causes deciliation [37,39].

A correlation between HIF-1alpha pathway activation and decreased early NLS was confirmed in the present study (Table 3). HIF-1α pathway activation occurs within the portal–parenchymal interface, the location of the progenitor cell niche, and can stimulate liver fibrogenesis, with detrimental effects on prognosis [17]. In this study, the CRs correlated with HIF-1α positivity but not with the postoperative prognosis after portoenterostomy.

### 4.5. Effects of Ciliary and Cytoplasmic TUBA4A Alterations on the Clinical–Laboratory Status and Native Liver Survival

#### 4.5.1. Effects on Clinical–Laboratory Status

Correlations were observed between the need for early LTx, decreased cilia length, and bilirubin serum levels (Table 3). Bilirubin serum levels at the time of portoenterostomy seemed to be affected by decreased cilia length, which can disturb the bile acids’ signaling pathways [35,40]. Additionally, increased direct-reacting bilirubin serum levels, a marker of cholestasis severity, as well as decreased cilia length correlated with the need for early LTx.

#### 4.5.2. Effects on Native Liver Survival

This study found correlations between ciliary and cytoplasmic tubulin features and the early postoperative prognosis following portoenterostomy. Decreased cilia length, ciliary bending, and increased cytoplasmic TUBA4A expression in the biliary cells reduced the span of NLS (Figure 5). The detrimental effects of the PC disruptions observed in the present study are understandable since PC regulate several signaling pathways crucial for liver function [35,40].

A unique finding of this study was an increased expression in BA patients of the cytoplasmic TUBA4A in cholangiocytes in comparison to the controls (Figure 6A), suggesting that increased cytoplasmic levels of TUBA4A in cholangiocytes correlate with poorer outcomes in BA patients. Tubulin, a fundamental constituent of the cellular cytoskeleton, is affected by post-translational modifications by diverse molecular factors [41], altering microtubule structures and their associated functions. Toxic compounds, such as alcohol, harm liver cells through tubulin cytoplasmic hyper-stabilization [42,43,44,45,46].

### 4.6. Limitations of This Study

The limitations of the present study include the small sample size of both BA and the controls. Biliary atresia and intrahepatic neonatal cholestasis are rare hepatobiliary diseases, and the study design of investigations concerning details of pathophysiology and clinical correlations may be affected by the sample size, thus requiring the performance of multicenter clinical studies or the use of experimental models. An additional aggravating factor, specifically concerning BA, is the existence of different clinical variants, which may affect the accuracy of results. Given these concerns, we have chosen to limit our BA sample to the isolated type and to bypass the sample size constraints by performing exploratory histopathological analyses. Histopathological studies involving digital image analyses enable the evaluation of a large series of microanatomic structures in each patient, making it possible to identify specific histopathological and morphometric patterns related to our subjects of interest. Thus, we used a convenience sample of patients who were cared for and followed in the clinical practice and conducted this exploratory histopathological analysis, being able to obtain statistically significant results with reasonable internal validity. Aiming to confirm the clinical effect of the statistically significant data, we determined the magnitude of the effect size developed by Cohen (1988). Our future next steps will include confirmatory experimental methods. Additionally, studies with larger sample sizes are necessary to extrapolate our findings to the population of BA patients, i.e., to ensure their external validity, not only for the isolated BA variant but also for the remaining clinical types.

## 5. Conclusions

**Figure 7:**
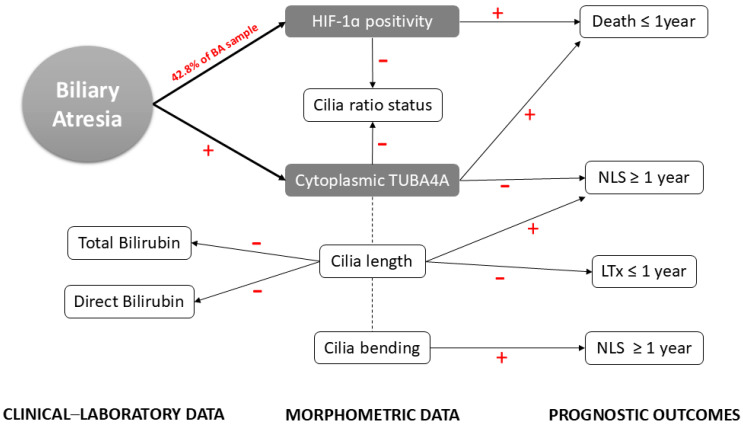
Overview of study findings. The flowchart represents the relationship between BA clinical–laboratory parameters, morphometric data, and prognostic outcomes analyzed in the present study, highlighting the role of PC dysfunction and HIF-1α activation in BA prognostic (+: directly proportional; −: inversely proportional).

This study evaluated the presence and clinical–laboratory outcomes attributable to alterations in the primary cilia and cytoplasmic tubulin expression in the intrahepatic biliary epithelium, observing that reduced PC length, decreased PC bending, and increased intensity of cytoplasmic TUBA4A occur in the isolated BA type, and negatively impact the postoperative prognosis after portoenterostomy (Figure 7). These findings suggest the existence of a disruption in the tubulin transport between cytoplasm and PC. The detrimental effect of the HIF-1alpha pathway activation on native liver survival was confirmed but unassociated with PC or cytoplasmic tubulin features.

## Figures and Tables

**Table 1:** Compared variables in the present study.

**Diagnostic (groups)** Isolated biliary atresia Intrahepatic neonatal cholestasis
**Clinical–laboratory** Age at portoenterostomy (days of life) Total and direct-reacting bilirubin serum levels at exploratory laparotomy
**Prognostic outcome evaluation** Age at liver transplantation (days of life) Age at death (days of life) Cause of liver transplantation Cause of death 1-year native liver survival
**Immunofluorescence features** ***Subjective (qualitative) method*** HIF-1alpha nuclear positivity in intrahepatic cholangiocytes ***Objective (quantitative, morphometric) methods *** ***(portal biliary structures; cells of the portal, or septal/parenchymal interface; and areas of ductular reaction distributed externally to parenchymal zone 1)*** ○ HIF-1alpha nuclear positivity (according to the positivity threshold of the control specimen) ○ TUBA4A positivity in primary cilia (according to the positivity threshold of the control specimen): Cilia ratio status (ratio between cilia count/number of bile ducts and ductules analyzed per patient) Cilia length Cilia bending ○ Cytoplasmic tubulin TUBA4A positivity (according to the positivity threshold of the control specimen) ○ DAPI-positive nuclei

**Table 2:** Demographic and clinical characteristics of patients and controls.

Patient ID	Diagnosis	Age PE (Days)	Age LTx (Days)	Age Death (Days)	Cause of LTx	Cause of Death	Total Bilirubin (mg/dL)	Direct Bilirubin (mg/dL)
BA1	Isolated Biliary Atresia	110	118	*Alive*	Liver Failure	*Alive*	9.5	7.5
BA2		56	*No LTx*	208	*No LTx*	Cirrhosis; liver failure; hemorrhage	7	5.4
BA3		48	*No LTx*	*Alive*	*No LTx*	*Alive*	7.8	3.5
BA4		54	*No LTx*	285	*No LTx*	Cirrhosis; liver failure	6.7	5.3
BA5		59	*No LTx*	283	*No LTx*	Cirrhosis; liver failure	5.8	4.2
BA6		93	*No LTx*	165	*No LTx*	Cirrhosis; liver failure	10	6.7
BA7		59	*No LTx*	*Alive*	*No LTx*	*Alive*	12	8.8
BA8		92	2559	*Alive*	Cirrhosis; liver failure	*Alive*	12.9	9.6
BA9		69	*No LTx*	*Alive*	*No LTx*	*Alive*	7.5	6
BA10		65	114	122	Liver failure	Cirrhosis; liver failure	19.1	14.3
BA11		52	212	*Alive*	Cirrhosis; liver failure	*Alive*	11.6	8.6
BA12		56	1409	*Alive*	Recurrent cholangitis; cirrhosis; liver failure	*Alive*	16.6	11.8
BA13		32	*No LTx*	*Alive*	*No LTx*	*Alive*	4.7	3.5
BA14		43	*No LTx*	*Alive*	*No LTx*	*Alive*	4.6	4
C1	Idiopathic Neonatal Cholestasis	35	*No LTx*	*Alive*	*No LTx*	*Alive*	14.6	10
C2		78	*No LTx*	*Alive*	*No LTx*	*Alive*	19.2	14.9
C3		62	*No LTx*	*Alive*	*No LTx*	*Alive*	12.1	8.8
C6	Parenteral Nutrition-Associated Cholestasis	62	*No LTx*	*Alive*	*No LTx*	*Alive*	14.6	10
C4	A1ATD	40	*No LTx*	*Alive*	*No LTx*	*Alive*	9.2	6.4
C5	CMV Hepatitis	35	*No LTx*	*Alive*	*No LTx*	*Alive*	*Not Available*	*Not Available*

**Table 3:** Spearman’s correlation between quantitative and qualitative clinical variables.

Variable Pair	Spearman’s Correlation (ρ)	*p*-Value	Significance
Clinical–laboratory status			
*Total Bilirubin* vs. *Cilia Length*	−0.665	0.013	*
*Direct Bilirubin* vs. *Cilia Length*	−0.758	0.003	**
Morphometric variables			
*Lower TUBA4A* vs. *CRs*	0.890	<0.001	**
Outcomes			
*LTx* vs. *Direct Bilirubin*	0.624	0.023	*
*LTx* vs. *Cilia Length*	−0.802	0.001	**
*Death* vs. *HIF-1α Positive Nuclei*	0.692	0.009	**
*Death* vs. *Higher TUBA4A*	0.626	0.022	*

Abbreviations: CRs—cilia ratio status; HIF-1α: hypoxia-inducible factor-1alpha in cholangiocytes; LTx—liver transplantation; TUBA4A—cytoplasmic alpha-tubulin 4 acetylated; PE—portoenterostomy; NLS—native liver survival. Death and Liver transplantation: represents the outcome until 1 year of life. Characterization of the type of variables under study: Qualitative variables: death; liver transplantation. Quantitative variables: bilirubin serum levels; cilia length; age; levels of TUBA4A and HIF-1α. In the table, * indicates *p*-values between 0.05 and >0.01, while ** indicates *p*-values ≤0.01, representing higher statistical significance.

## Data Availability

The original contributions presented in the study are included in the article/Supplementary Material, further inquiries can be directed to the corresponding author.

## Supplementary Materials

The following supporting information can be downloaded at https://www.mdpi.com/article/10.3390/biomedicines13010087/s1, Figure S1: Positive controls for primary antibodies. (A) Human gallbladder stained for HIF1alpha (yellow). (B) Human testicle stained for TUBA4A (red); Figure S2: Representative images illustrating the microanatomical structures and staining patterns analyzed in this study; Figure S3: Schematic resume of CiliaQ plugin workflow; Table S1: Cohen’s effect size. Reference [47] is cited in the supplementary materials.
