# Supplementary material for: Structural Disruption of Cilia and Increased Cytoplasmic Tubulin in Biliary Atresia—An Exploratory Study Focusing on Early Postoperative Prognosis Following Portoenterostomy
Source: Biomedicines. 2025 Jan 1;13(1):87. doi: 10.3390/biomedicines13010087 (PMC11763231; doi:10.3390/biomedicines13010087)
Supplement: Supplementary file 1 [file biomedicines-13-00087-s001.zip › biomedicines-3302427-supplementary.pdf]

Supplemental Material

IMAGES

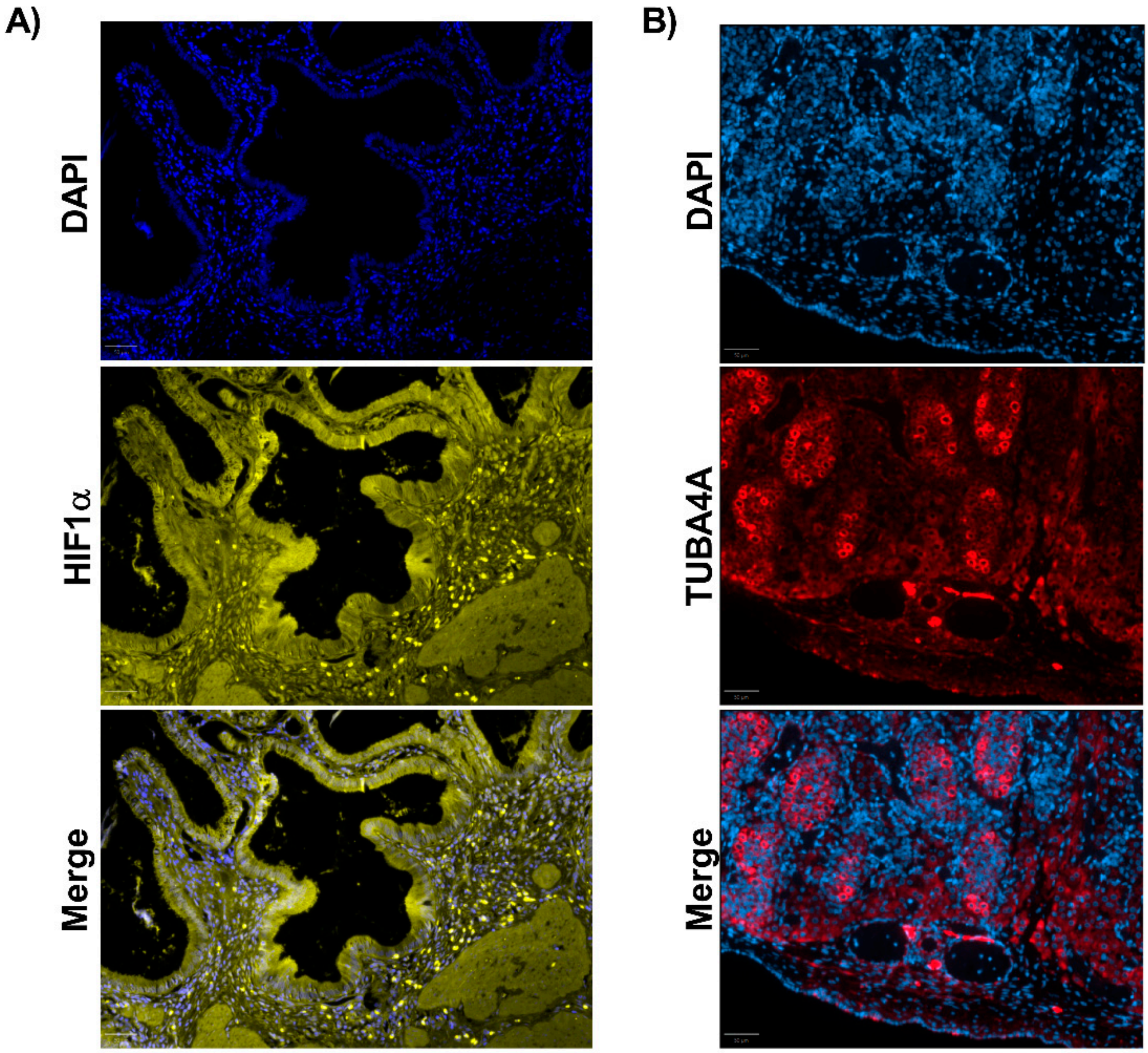

Figure S1. Positive controls for primary antibodies. A) Human gallbladder stained for HIF-1α (yellow). B) Human testicle stained for TUBA4A (red). Blue: Dapi ; Red: TUBA4A ; Yellow: HIF-1α . Images magnification: 40x

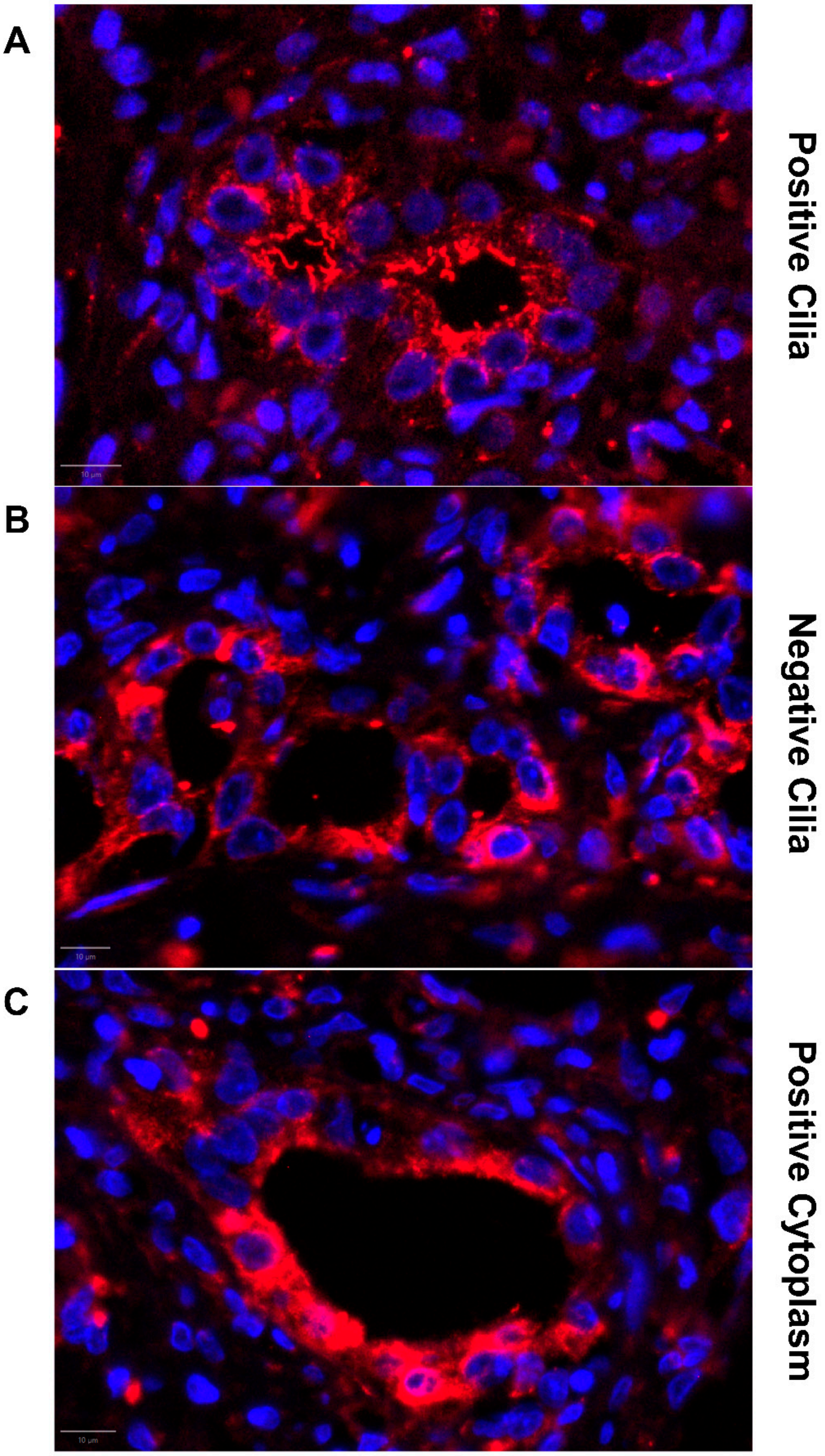

Figure S2. Representative images illustrating the microanatomical structures and staining patterns analyzed in this study. These images highlight key features relevant to the investigation, showcasing the structural organization and specific staining used for detailed analysis. A) Representative biliary duct presenting cilia in cholangiocytes; B) Representative biliary duct without cilia in cholangiocytes; C) Representative biliary duct with cholangiocytes positive for Tubulin in cytoplasm (Blue – DAPI; Red – TUBA4A, Magnification: 63x)

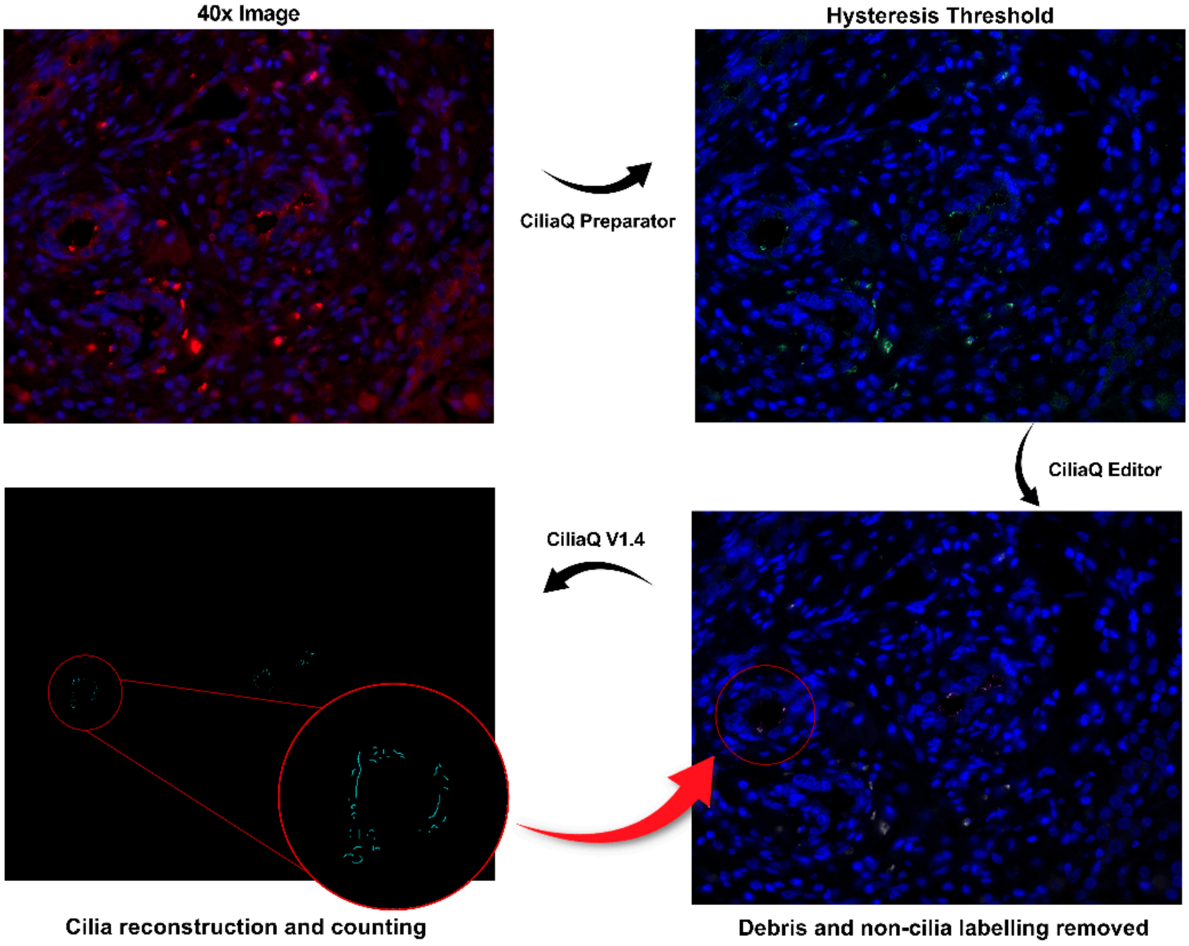

Figure S3. Schematic resume of CiliaQ plugin workflow. Blue – Dapi; Red – TUBA4A; Green – Processed Cilia by the program; Purple: Final cilia, after debris removed. Magnification: 40x.

TABLES

Table S1. ***Cohen's effect size.*** Interpretation of the effect size statistics according to Hopkins (2002)

**Magnitude of the difference**	**Effect size (d Cohen)**
Trivial	0.001 |—
Small	0,2 |—
Moderate	0,6 |—
Large	1,2 |—
Very large	2,0 |—
Nearly perfect	4,0 |—
Perfect	∞ |—

Reference 47: Cohen, J. (1988). Statistical power analysis for the behavioral sciences (2nd ed.). New Jersey: Lawrence Erlbaum.
